# Silencing of long non-coding RNA Sox2ot inhibits oxidative stress and inflammation of vascular smooth muscle cells in abdominal aortic aneurysm via microRNA-145-mediated Egr1 inhibition

**DOI:** 10.18632/aging.103077

**Published:** 2020-07-06

**Authors:** Huyu Lin, Bin You, Xiandong Lin, Xiaohu Wang, Dongsheng Zhou, Zhiqun Chen, Yuanxiang Chen, Ren Wang

**Affiliations:** 1 Department of Cardiovascular Surgery, Provincial Clinical College of Fujian Medical University, Fujian Provincial Hospital, Fuzhou 350001, P.R. China; 2 Department of Cardiology, Provincial Clinical College of Fujian Medical University, Fujian Provincial Hospital, Fuzhou 350001, P.R. China; 3 Department of Imaging, Provincial Clinical College of Fujian Medical University, Fujian Provincial Hospital, Fuzhou 350001, P.R. China

**Keywords:** long non-coding RNA Sox2ot, MicroRNA-145, Egr1, abdominal aortic aneurysm, oxidative stress

## Abstract

Long non-coding RNAs (lncRNAs) have been largely reported to contribute to the development and progression of abdominal aortic aneurysm (AAA), a common vascular degenerative disease. The present study was set out with the aim to investigate the possible role of lncRNA Sox2ot in the development of AAA. In this study, we found that lncRNA Sox2ot and early growth response factor-1 (Egr1) were highly expressed, while microRNA (miR)-145 was poorly expressed in Ang II-induced AAA mice and oxidative stress-provoked vascular smooth muscle cell (VSMC) model. Egr1 was a potential target gene of miR-145, and lncRNA Sox2ot could competitively bind to miR-145 to upregulate Egr1 expression. Overexpression of miR-145-5p was found to attenuate oxidative stress and inflammation by inhibiting Egr1 both *in vivo* and *in vitro*, which was counteracted by lncRNA Sox2ot. Taken together, the present study provides evidence that downregulation of lncRNA Sox2ot suppressed the expression of Egr1 through regulating miR-145, thus inhibiting the development of AAA, highlighting a theoretical basis for AAA treatment.

## INTRODUCTION

Being the most common types of aortic aneurysm, abdominal aortic aneurysm (AAA) is regarded as an age-related degenerative disease and characterized by the permanent enlargement of the lower abdominal aorta [1, 2]. The incidence of AAA in males over the age of 65 is 4-7% and in females, it is 1-2% [3]. Moreover, pathological processes, such as oxidative stress, cell apoptosis, and inflammation are implicated in the development of AAA [4]. Notably, vascular smooth muscle cells (VSMCs) are known as major components of the arterial wall, however, their dysfunction results in increased oxidative stress and inflammation which has been reported to play a vital role in AAA [5]. For instance, being one of the pathological causes of AAA, oxidative stress in VSMCs can lead to the degeneration of aortic walls and eventual rupture [6]. Besides, apoptosis is another pathological characteristics of VSMCs, reported to be involved in the AAA progression [3]. To date, surgical management is considered as one of the main therapies for AAA, yet, the mortality of AAA remained very high [7]. Thus, it is urgent to identify novel therapeutic strategies against the pathological causes of AAA, particularly, regarding oxidative stress and inflammation in VSMCs.

Long noncoding RNAs (lncRNAs) are a group of transcripts, characterized by the length exceeding 200 nucleotides and significantly lack the protein-coding potential [8]. Yang et al. have demonstrated the effects of lncRNAs on the pathogenesis of AAA [9]. LncRNA Sox2 overlapping transcript (Sox2ot) has been reported to locate at human chromosome 3q26.33. Moreover, LncRNA Sox2 has been attributed to the pathophysiological progression of human cancer due to its amplification in lung squamous cell carcinomas (SCCs) [10]. Of note, accumulating studies have reported the involvement of lncRNA Sox2ot in the progression of breast cancer [11]. However, the functional role of lncRNA Sox2ot in these tumors remained to be elucidated. Notably, up till now, scarce studies are determining the role of lncRNA Sox2ot in oxidative stress and inflammatory VSMCs in AAA.

Intriguingly, MicroRNAs (miRNAs) are known as short non-coding RNAs with 18-22 nucleotides with the ability to regulate the target gene expression [12]. Peculiarly, miR-21 could modulate the cardiovascular pathology and function as the therapeutic option to inhibit the AAA progression [13]. Noticeably, miR-145, a 22 nucleotide long and highly conserved miRNA, has been reported as a potential target for the progression of AAA [2, 14]. Importantly, by using this bioinformatics prediction in combination with a dual-luciferase reporter gene, here we confirmed that early growth response factor-1 (Egr1) is a potential target gene of miR-145. Nevertheless, the conducive role of Egr1 in thrombogenic and inflammatory reaction has been well established in AAA [15]. Whilst Egr1, a zinc finger transcription factor and a member of the early gene family, has been indicated to participates in various pathophysiological processes, such as cell proliferation, apoptosis, and inflammatory response [16]. Though the roles of lncRNA Sox2ot, miR-145-5p and Egr1 in the progression of AAA have been reported respectively, the deeper understanding of the effects of the lncRNA Sox2ot/miR-145/Egr1 signaling axis on AAA remains unclear. Therefore, this present study aimed to investigate the role of the newly discovered lncRNA Sox2ot in the oxidative stress response and inflammation of VSMCs in AAA, to provide a novel direction for AAA treatment.

## RESULTS

### GEO bioinformatics analysis predicts downregulated miR-145 in mice with Ang II-induced AAA

**Figure 1:**
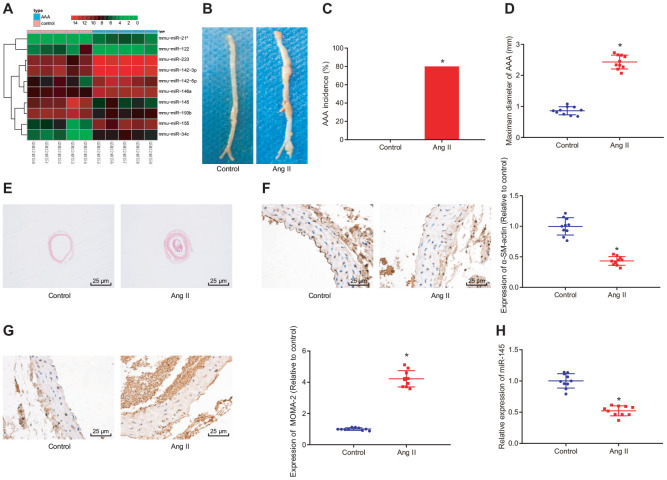
**GEO bioinformatics analysis predicting poorly expressed miR-145 in mice with Ang II-induced AAA.** (**A**) a heatmap of the top 10 differentially expressed miRNAs obtained from the AAA-related microarray data GSE51226 downloaded from the Gene Expression Omnibus (GEO) database (https://www.ncbi.nlm.nih.gov/geo/); the abscissa represents sample number and the ordinate represents names of miRNAs; each small square in the figure represents the expression level of a miRNA in one sample, and the histogram in the upper right represents color grading; (**B**) representative images of the morphology of abdominal aorta specimens of the control ApoE^-/-^ mice and Ang II-induced AAA ApoE^-/-^ mice; (**C**) incidence of AAA in ApoE^-/-^ mice; (**D**) the maximum diameter of abdominal aorta in mice; (**E**) morphological changes of abdominal aorta in mice observed by HE staining (× 400); (**F**) α-SM-actin expression in SMCs in abdominal aorta determined using immunohistochemistry (× 400); (**G**) MOMA-2 expression in monocyte and SMCs in abdominal aorta determined using immunohistochemistry (× 400); (**H**) miR-145 expression measured using RT-qPCR; * *p* < 0.05 compared with ApoE^-/-^ mice; measurement data were depicted as the mean ± standard deviation; comparisons between the two groups were analyzed using an unpaired t-test; n = 10.

We first performed a differential miRNA expression analysis on an AAA-related expression dataset GSE51226 with the |log2 FoldChange (FC)| > 1.0 and *adj.P.Val* < 0.05 set as a threshold. A heat map illustrating the top 10 differentially expressed miRNAs is shown in Figure 1A, which showed that mmu-miR-145 was the most significantly downregulated miRNA in AAA (logFC = -4.16). In ApoE^-/-^ mice, the incidence of AAA induced by Ang II was about 80%. The abdominal aorta of the Ang II-induced AAA mice was enlarged, with the maximum diameter close to 2.5 mm, which was significantly larger than that of ApoE^-/-^ mice (Figure 1B–1D). Interestingly, this phenomenon was more prominent in hematoxylin-eosin (HE) staining images (Figure 1E), suggesting that Ang II-induced AAA mouse models were successfully established. Furthermore, immunohistochemistry results showed that Ang II induction significantly reduced the SMC content in the abdominal aortic wall and enhanced the macrophage content in mice (*p* < 0.05) (Figure 1F, 1G). Meanwhile, reverse transcription-quantitative polymerase chain reaction (RT-qPCR) results indicated poorly expressed miR-145 in the abdominal aorta of the Ang II-induced AAA mice (*p* < 0.05) (Figure 1H), which was consistent with the results from the aforementioned bioinformatics analysis. These findings demonstrated that miR-145 could play a vital role in the progression of AAA.

### Upregulation of miR-145 inhibits the occurrence and progression of AAA in ApoE^-/-^ mice

**Figure 2:**
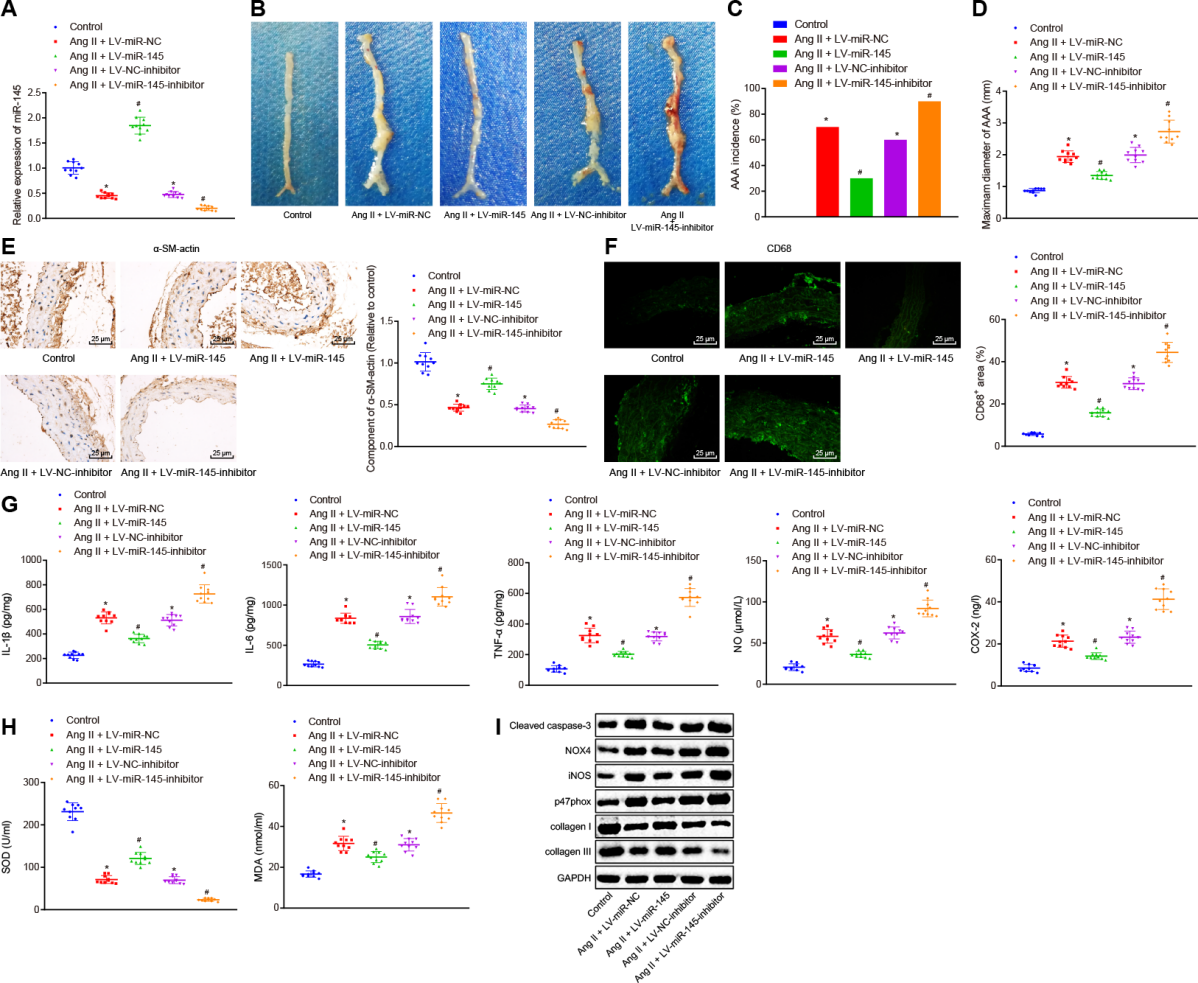
**miR-145 suppresses the occurrence and progression of AAA in ApoE^-/-^ mice.** (**A**) interference efficiency of miR-145 verified by RT-qPCR; (**B**) representative morphology images of abdominal aorta specimens in mice; (**C**) incidence of AAA in mice; (**D**) the maximum diameter of abdominal aorta in mice; (**E**) α-SM-actin expression in SMCs in abdominal aorta determined using immunohistochemistry (× 400); (**F**) CD68 expression in abdominal aorta detected using immunofluorescence staining (× 400); (**G**) levels of COX-2, NO, IL-1β, IL-6 and TNF-α in serum of mice measured using ELISA; (**H**) SOD level in serum and MDA level in abdominal aorta of mice; (**I**) protein levels of cleaved caspase-3, NOX4, iNOS, p47phox, collagen I and collagen III determined using Western blot analysis; * *p* < 0.05, *vs.* normal mice; # *p* < 0.05, *vs.* AAA mice injected with LV-miR-NC or LV-NC-inhibitor plasmids; measurement data were depicted as the mean ± standard deviation; comparisons among multiple groups were analyzed using one-way ANOVA followed by Turkey’s post hoc test; n = 10.

To further investigate the effects of miR-145 on the progression of AAA, mice were injected with the corresponding recombinant lentiviruses carrying LV-miR-NC, LV-miR-145, LV-negative control (NC)-inhibitor, and LV-miR-145-inhibitor, respectively, one day after induction of Ang II to ApoE^-/-^ mice. The abdominal aorta of mice was extracted for analysis. Our results suggested that recombinant lentiviruses were constructed successfully (*p* < 0.05) (Figure 2A). Moreover, we found that compared with the normal mice, AAA mice injected with LV-miR-NC and LV-NC-inhibitor exhibited increased AAA incidence and the maximum diameter of abdominal aorta. AAA mice with overexpression of miR-145 exhibited significantly reduced AAA incidence and maximum diameter of abdominal aorta in comparison to AAA mice injected with LV-miR-NC. Accordingly, opposite trends were observed when miR-145 was down-regulated by injecting AAA mice with LV-miR-145-inhibitor in comparison to those injected with LV-NC-inhibitor (*p* < 0.05) (Figure 2B–2D).

The changes of SMC and macrophages in abdominal aorta of AAA mice were further assessed by immunohistochemistry and immunofluorescence assay with the contents of alpha-smooth muscle actin (α-SM-actin) and CD68. Our results showed that SMC was decreased and macrophages were accumulated in abdominal aorta in AAA mice injected with LV-miR-NC and LV-NC-inhibitor. Compared with the AAA mice injected with LV-miR-NC, AAA mice injected with LV-miR-145 exhibited elevated SMC but decreased the macrophages. Similarly, AAA mice injected with LV-miR-145-inhibitor demonstrated opposite results to those of the abovementioned overexpression of miR-145 in comparison to AAA mice injected with LV-NC-inhibitor (*p* < 0.05) (Figure 2E, 2F).

Furthermore, enzyme-linked immunosorbent assay (ELISA) was adapted to measure the levels of inflammatory factors and oxidative stress-related factors, such as nitric oxide (NO), superoxide dismutase (SOD), and malonaldehyde (MDA) in serum and abdominal aorta of mice. The findings of ELISA proved that compared with the normal mice, AAA mice injected with LV-miR-NC and LV-NC-inhibitor showed increased levels of cyclooxygenase 2 (COX-2), NO, interleukin (IL)-1β, IL-6, tumor necrosis factor (TNF)-α, MDA, cleaved caspase-3, NADPH oxidase 4 (NOX4), inducible nitric oxide synthase (iNOS), and p47phox, however, decreased levels of SOD, collagen I, and collagen III were observed, respectively, indicating that levels of inflammation and oxidative stress in AAA mice were higher than those in normal mice. Compared with AAA mice injected with LV-miR-NC, AAA mice injected with LV-miR-145 showed reduced levels of COX-2, NO, IL-1β, IL-6, TNF-α, MDA, cleaved caspase-3, NOX4, iNOS, and p47phox, whereas increased levels of SOD, collagen I, and collagen III were observed. On the other hand, AAA mice injected with LV-miR-145-inhibitor indicated the opposite results in comparison to AAA mice injected with LV-NC-inhibitor (*p* < 0.05) (Figure 2G–2I). Thus, we concluded that miR-145 could inhibit the occurrence and progression of AAA by reducing inflammation and oxidative stress in ApoE^-/-^ mice.

### Upregulation of miR-145 inhibits apoptosis, inflammatory reaction, and oxidative stress in Ang II-treated VSMCs

**Figure 3:**
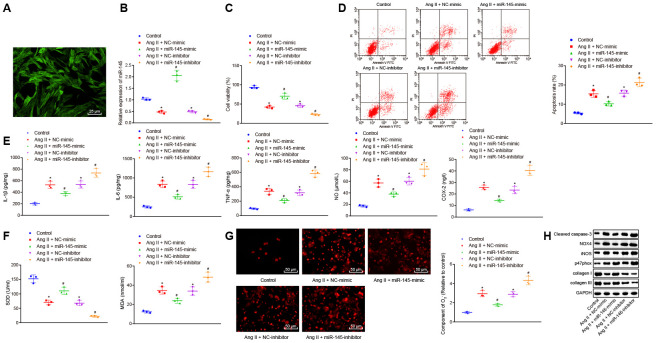
**miR-145 suppresses the apoptosis, inflammatory reaction, and oxidative stress of VSMCs treated with Ang II.** (**A**) the expression of the molecular marker α-SM-actin of VSMCs isolated by immunofluorescence assay (× 400); (**B**) miR-145 expression in VSMCs determined using RT-qPCR; (**C**) viability of VSMCs detected by CCK-8 assay; (**D**) apoptosis of VSMCs detected by flow cytometry; (**E**) levels of COX-2, NO, IL-1β, IL-6, and TNF-α in serum of VSMCs measured by ELISA; (**F**) levels of MDA and SOD in serum of VSMCs measured using kits; (**G**) O_2_^-^ in VSMCs measured using DHE staining (× 400); (**H**) protein levels of cleaved caspase-3, NOX4, iNOS, p47phox, collagen I and collagen III in VSMCs determined using Western blot analysis; *, *p* < 0.05, *vs.* VSMCs without treatment; #, *p* < 0.05, *vs.* VSMCs treated with Ang II + LV-miR-NC or Ang II+LV-NC-inhibitor plasmids; measurement data were depicted as the mean ± standard deviation; comparisons among multiple groups were analyzed using one-way ANOVA followed by Turkey’s post hoc test; the experiment was repeated three times.

After determining the roles of miR-145 in inflammation and oxidative stress in Ang II-induced AAA mice, we further aimed to explore the role of miR-145 in VSMCs. According to the method from a recent study [1], we established an oxidative-stressed VSMC model and then interfered with the expression of miR-145. Initially, immunofluorescence staining results exhibited the abundant green filaments in the cytoplasm of the isolated VSMCs, indicating the successful isolation of VSMCs with 99% purity index (Figure 3A). Besides, RT-qPCR results showed that miR-145 expression was significantly reduced in Ang II-induced VSMCs overexpressing miR-145 while it was downregulated in the absence of miR-145 (*p* < 0.05) (Figure 3B).

Cell counting kit-8 (CCK-8) assay and flow cytometry demonstrated that compared with the VSMCs without treatment, cell viability was inhibited and apoptosis was promoted in response to treatment with NC-mimic and NC-inhibitor plasmids. The upregulation of miR-145 increased the viability and inhibited the apoptosis of Ang II-induced VSMCs, which was abrogated by miR-145 downregulation (*p* < 0.05) (Figure 3C, 3D). Meanwhile, Ang II-induced VSMCs treated with NC-mimic or NC-inhibitor displayed elevated levels of IL-1β, IL-6, TNF-α, COX-2, NO, MDA, O_2_^-^, caspase-3, NOX4, iNOS, p47phox whereas reduced levels of SOD, collagen I, and collagen III were observed, in comparison to untreated VSMCs. Ang II-induced VSMCs treated with miR-145-mimic plasmids showed decreased levels of IL-1β, IL-6, TNF-α, COX-2, NO, MDA, O_2_^-^, caspase-3, NOX4, iNOS, p47phox, however, elevated levels of SOD, collagen I, and collagen III were observed, when compared with Ang II-induced VSMCs treated with NC-mimic. Additionally, Ang II-induced VSMCs treated with miR-145-inhibitor showed a significant increase in the levels of IL-1β, IL-6, TNF-α, COX-2, NO, MDA, O_2_^-^, caspase-3, NOX4, iNOS, p47phox whereas a decline in levels of SOD, collagen I, and collagen III was observed, in comparison to NC-inhibitor-treated VSMCs after Ang II induction (*p* < 0.05) (Figure 3E–3H). Taken together, these results demonstrated that elevated miR-145 could suppress the apoptosis, inflammatory reaction, and oxidative stress in the VSMCs from Ang II-induced AAA mice.

### LncRNA Sox2ot competitively binds to miR-145

**Figure 4:**
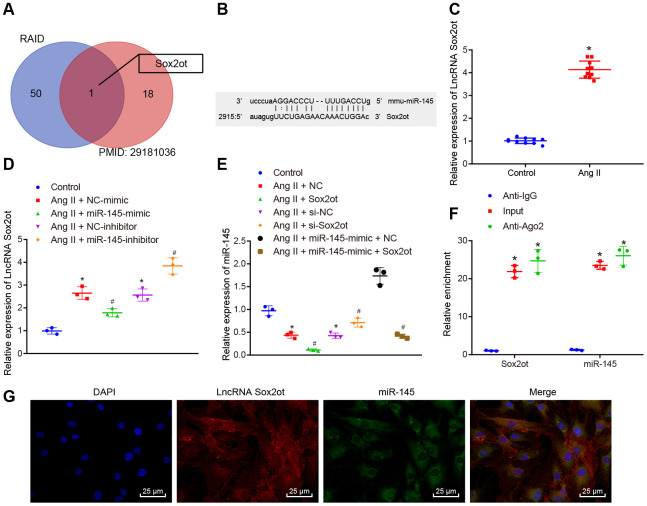
**miR-145 is negatively regulated by lncRNA Sox2ot.** (**A**) comparisons of predicted lncRNAs that target miR-145 in RAID database (http://www.rna-society.org/raid/index.html) and lncRNAs related to AAA reported in a prior study; (**B**) Sequence complementarity between lncRNA Sox2ot and miR-145 analyzed by RNA22; (**C**) lncRNA Sox2ot expression in abdominal aorta of normal mice and Ang II-induced AAA mice determined using RT-qPCR; (**D**) lncRNA Sox2ot expression in VSMCs determined by RT-qPCR; (**E**) miR-145 expression in VSMCs measured by RT-qPCR; (**F**) RIP assay with anti-Ago2, IgG, or 10% input from VSMC extracts. RNA levels in the immunoprecipitates were determined by RT-qPCR; (**G**) co-localization of miR-145 and lncRNA Sox2ot detected by FISH (× 400); * *p* < 0.05, *vs.* VSMCs without treatment; # *p* < 0.05, *vs.* VSMCs treated with Ang II + LV-miR-NC or Ang II + LV-NC-inhibitor plasmids; measurement data were depicted as the mean ± standard deviation; comparisons between the two groups were analyzed using an unpaired t-test, and comparisons among multiple groups were analyzed using one-way ANOVA, followed by Turkey’s post hoc test; each experiment was repeated three times.

To understand the upstream regulatory mechanism of miR-145, the potential lncRNAs that interact with miR-145 were initially predicted using the RAID (http://www.rna-society.org/raid/) dataset, with 51 lncRNAs obtained. Moreover, bioinformatics analysis by Zhou et al. [8] has reported the top 19 lncRNAs related to AAA, which were further intersected with the predicted lncRNAs from the RAID database using Venn diagram. LncRNA Sox2ot was finally obtained (Figure 4A). Subsequently, the subcellular localization of lncRNA Sox2ot was predicted using the lncLocator database website: (http://www.csbio.sjtu.edu.cn/bioinf/lncLocator/), based on its sequence obtained from National Center for Biotechnology Information (NCBI), LncRNA Sox2ot was predicted to be localized in the cytoplasm (89.67%). Therefore, we were convinced that the expression of miR-145 might be regulated by Sox2ot in AAA. Moreover, the RNA22 website (https://cm.jefferson.edu/rna22/Interactive/) was used to predict the binding site between lncRNA Sox2ot and miR-145, with complementary binding sites found between these two sequences (Figure 4B). At the same time, we examined the expression level of lncRNA Sox2ot in abdominal aorta of normal mice and AAA mice by RT-qPCR. The results showed that lncRNA Sox2ot was highly expressed in AAA mice, which was contrary to the expression pattern of miR-145 (*p* < 0.05) (Figure 4C). Furthermore, the expression of lncRNA Sox2ot was decreased in Ang II-induced VSMCs overexpressing miR-145 while it was significantly increased upon miR-145 knockdown (*p* < 0.05) (Figure 4D), suggesting that lncRNA Sox2ot could be negatively regulated by miR-145. Meanwhile, in Ang II-induced VSMCs, overexpressed or inhibited lncRNA Sox2ot significantly reduces or increases the miR-145 expression, respectively (*p* < 0.05) (Figure 4E), indicating that lncRNA Sox2ot could also negatively regulate miR-145. Additionally, RNA binding protein immunoprecipitation (RIP) assay was conducted to further verify the negative correlation between lncRNA Sox2ot and miR-145, the results of which demonstrated that lncRNA Sox2ot and miR-145 were preferentially enriched in Argonaute2 (Ago2)-containing beads in VSMCs, relative to control IgG (*p* < 0.05) (Figure 4F). Moreover, fluorescence in situ hybridization (FISH) results also showed that miR-145 co-localized with lncRNA Sox2ot in the cytoplasm of mouse VSMCs (*p* < 0.05) (Figure 4G). Overall, the obtained data suggested that lncRNA Sox2ot could bind to miR-145 and negatively regulate its expression.

### LncRNA Sox2ot reverses the inhibitory roles of miR-145 in oxidative stress and inflammation of Ang II-treated VSMCs

**Figure 5:**
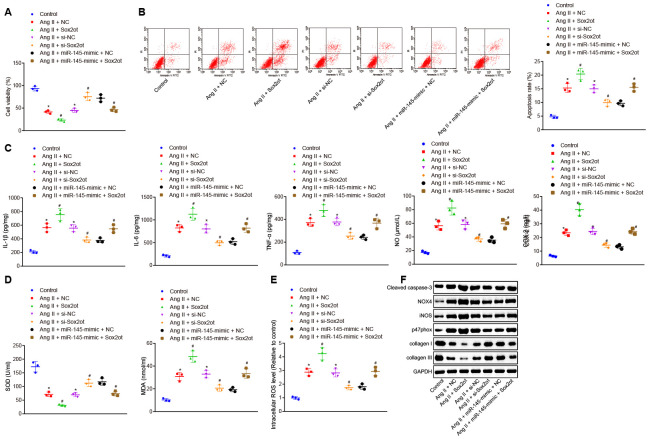
**LncRNA Sox2ot reverses the inhibitory roles of miR-145 in oxidative stress and inflammation of VSMCs treated with Ang II.** (**A**) viability of VSMCs detected by CCK-8 assay; (**B**) apoptosis of VSMCs detected by flow cytometry; (**C**) levels of COX-2, NO, IL-1β, IL-6, and TNF-α in serum of VSMCs measured by ELISA; (**D**) levels of MDA and SOD in serum of VSMCs measured using kits; (**E**) ROS level in VSMCs measured using kits; (**F**) protein levels of cleaved caspase-3, NOX4, iNOS, p47phox, collagen I and collagen III determined using Western blot analysis; * *p* < 0.05, *vs.* VSMCs without treatment; # *p* < 0.05, *vs.* VSMCs treated with Ang II + NC-mimic, Ang II + NC-inhibitor, and Ang II + miR-145-mimic + NC plasmids; measurement data were depicted as the mean ± standard deviation; comparisons among multiple groups were analyzed using one-way ANOVA followed by Turkey’s post hoc test; the experiment was repeated three times.

After determining the interaction between lncRNA Sox2ot and miR-145, the role of lncRNA Sox2ot in VSMCs from AAA mice and the mechanism of its effect on cellular oxidative stress and inflammation were further explored. CCK-8 assay and flow cytometry demonstrated that compared with Ang II-induced VSMCs treated with NC or small interfering RNA (si)-NC plasmids, Ang II-induced cells treated with Sox2ot plasmids indicated decreased cell viability and increased apoptosis, while Ang II-induced cells treated with si-Sox2ot exhibited opposite results. In consent, with our study that lncRNA Sox2ot could negatively regulate miR-145. Moreover, the cell viability and apoptosis of VSMCs from induced AAA mice that were treated together with both Sox2ot and miR-145-mimic plasmids were analyzed. Compared with the Ang II-induced cells treated with miR-145-mimic + NC plasmids, Ang II-induced cells treated with miR-145-mimic + Sox2ot plasmids exhibited inhibited cell viability and enhanced apoptosis (*p* < 0.05) (Figure 5A, B), suggesting that lncRNA Sox2ot could reverse the roles of miR-145 in Ang II-induced VSMCs. ELISA results implied that overexpressed lncRNA Sox2ot aggravated the oxidative stress and inflammation of VSMCs treated with Ang II, which were reflected by the increased levels of reactive oxygen species (ROS), COX-2, NO, IL-1β, IL-6, TNF-α, and MDA, and the decreased SOD levels. Silencing lncRNA Sox2ot could relive oxidative stress and inflammation of VSMCs treated with Ang II, which were manifested by reduced levels of ROS, COX-2, NO, IL-1β, IL-6, TNF-α, and MDA, whereas SOD level was elevated. Furthermore, the inhibitory effect of miR-145 on oxidative stress and inflammation was negated by dual treatment with miR-145 mimic and lncRNA Sox2ot in Ang II-treated VSMCs (*p* < 0.05) (Figure 5C–5E). Over-expressed lncRNA Sox2ot increased the levels of cleaved caspase-3, NOX4, iNOS, and p47phox, whereas decreased levels of collagen I and collagen III were observed in Ang II-induced VSMCs. Moreover, repressed lncRNA Sox2ot resulted in correspondingly opposite results. The co-transfection of lncRNA Sox2ot and miR-145 plasmids counteract the effects of the miR-145 overexpression alone (*p* < 0.05) (Figure 5F). Thus, we speculated that lncRNA Sox2ot reversed the suppressive effects of miR-145 on oxidative stress and inflammation of the Ang II-induced VSMCs.

### LncRNA Sox2ot binding to miR-145 affects oxidative stress and inflammation of Ang II-treated VSMCs by regulating Egr1

**Figure 6:**
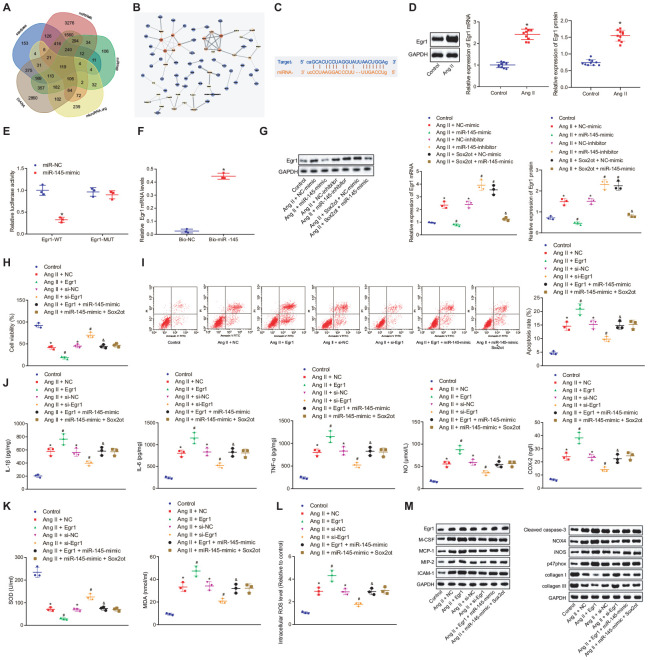
**Silencing of lncRNA Sox2ot binding miR-145 inhibits oxidative stress and inflammation of Ang II-treated VSMCs by downregulating Egr1.** (**A**) Venn map for 119 intersection genes predicted to be regulated by miR-145 in the DIANA (http://diana.imis.athena-innovation.gr/DianaTools/index.php?r=microT_CDS/index), miRWalk (http://mirwalk.umm.uni-heidelberg.de/), miRmap (https://mirmap.ezlab.org/), microRNA.org (http://www.microrna.org/microrna/home.do), and starBase (http://starbase.sysu.edu.cn/index.php); (**B**) a PPI network using Cytoscape 3.6.0 software for target genes of miR-145 was constructed using string database (https://string-db.org/); the color represents the correlation between miR-145 and other genes, and red represent the high correlation; (**C**) the complementary binding sites between miR-145 and Egr1 3'-UTR predicted by StarBase; (**D**) Egr1 mRNA and protein levels in the abdominal aorta of normal mice and Ang II-induced AAA mice determined by RT-qPCR and Western blot analysis; (**E**) interaction between miR-145 and Egr1 detected by dual luciferase reporter gene assay; (**F**) the interaction between miR-145 and Egr1 verified by RNA pull down assay; (**G**) Egr1 mRNA and protein levels in VSMCs determined by RT-qPCR and Western blot analysis; (**H**) cell viability of VSMCs detected by CCK-8 assay; (**I**) apoptosis of VSMCs detected by flow cytometry; (**J**) levels of COX-2, NO, IL-1β, IL-6, and TNF-α in serum of VSMCs Egr1 and/or Sox2ot/miR-145 measured by ELISA; (**K**) levels of MDA and SOD in serum of VSMCs measured using kits; (**L**) ROS level in VSMCs measured by kits; (**M**) protein levels of cleaved caspase-3, NOX4, iNOS, p47phox, collagen I and collagen III in VSMCs determined using Western blot analysis; * *p* < 0.05, *vs.* normal mice or VSMCs without treatment; # *p* < 0.05, *vs.* VSMCs treated with Ang II + NC-mimic, Ang II + NC-inhibitor, Ang II + NC, or Ang II + si-NC plasmids; & *p* < 0.05, *vs.* VSMCs treated with Ang II + NC-mimic or Ang II + Egr1 plasmids; measurement data were depicted as the mean ± standard deviation; comparisons between the two groups were analyzed using unpaired t-test (n = 10), and comparisons among multiple groups were analyzed using one-way ANOVA, followed by Turkey’s post hoc test; the experiment was repeated three times.

The DIANA, miRWalk, miRmap, microRNA.org, and starBase databases were adopted to predict the target genes of miR-145, with 7009 and 1806 target genes found in the DIANA and starBase databases, as well as 6354 target genes 3’ untranslated region (UTR) found in the miRWalk database. Further screening according to their miRmap score and mirSVR score, 1785 target genes were found with miRmap score > 70 and 1103 target genes with mirSVR score < -0.5. At last, the differences of these target genes were analyzed by plotting a Venn diagram (Figure 6A), which demonstrated that there were 119 intersection genes, indicating the possibility to be regulated by miR-145. These genes were then incorporated into the String database to analyze gene interaction, and then a protein-protein interaction (PPI) network was constructed (Figure 6B). Among the 119 intersection genes, Cdh1, Egr1, and Synj1 were the top 3 highly interacted genes, and they were at the core of the network. Thus, we speculated that these three genes might affect the occurrence of disease. Meanwhile, a relevant study has shown that high expression of Egr1 was associated with thrombosis in human AAA [15]. Taken together, we speculated that miR-145 might affect AAA by targeting Egr1. To verify the hypothesis, we used the starBase database website, which predicted binding sites between miR-145 and Egr1 3'-UTR (Figure 6C). RT-qPCR and Western blot analysis revealed that Egr1 was highly expressed in abdominal aorta of Ang II-induced AAA mice (*p* < 0.05) (Figure 6D), which was opposite to the effect of miR-145 on AAA. Additionally, the results of dual-luciferase reporter gene assay showed that the luciferase activity of wild type (wt)-Egr1 3’-UTR was decreased significantly in HEK-293T cells transfected with miR-145-mimic plasmids (*p* < 0.05) (Figure 6E), suggesting that miR-145 was specifically bound to Egr1 3'-UTR. Meanwhile, the miR-145 probe could specifically enrich Egr1 mRNA (*p* < 0.05) (Figure 6F). These findings demonstrated that miR-145 could target and regulate Egr1.

Compared with the VSMCs without treatment, Ang II-induced VSMCs treated with NC-mimic and NC-inhibitor plasmids showed increased Egr1 level, which suggested that oxidative stress can stimulate the expression of Egr1 protein in VSMCs. Additionally, Ang II-induced VSMCs treated with miR-145-mimic plasmids indicated decreased Egr1 expression, which was reversed in Ang II-induced cells treated with miR-145-inhibitor plasmids. Dual treatment with Sox2ot and miR-145-mimic plasmids in Ang II-induced cells diminished the Egr1 expression compared with Ang II-induced cells treated with Sox2ot + NC-mimic plasmids (*p* < 0.05) (Figure 6G). Additionally, cell viability was decreased while cell apoptosis was enhanced in Ang II-treated cells relative to untreated cells. Ang II + Egr1 treatment lowered the cell viability and increased cell apoptosis, which was negated by Ang II + si-Egr1 treatment (*p* < 0.05). However, no significant changes were detected in cells treated with Ang II + Egr1 + miR-145-mimic, Ang II + miR-145-mimic + Sox2ot and Ang II + NC (*p* < 0.05) (Figure 6H, 6I). These results suggested that miR-145 could negatively regulate Egr1 and lncRNA Sox2ot could affect Egr1 level by regulating miR-145, thus affecting the survival efficiency of VSMCs induced by Ang II.

Besides, ELISA results revealed that overexpressed Egr1 could aggravate the oxidative stress and inflammation of VSMCs reflected by the increased levels of ROS, COX-2, NO, IL-1β, IL-6, TNF-α, and MDA along with decreased SOD levels. Vice versa, the inhibition of Egr1 displayed reverse trends. Treatment with both overexpressed Egr1 and miR-145 counteracted the effect of overexpressed Egr1 alone on oxidative stress and inflammation in Ang II-treated VSMCs, which was consistent with the results of simultaneously upregulated Sox2ot and miR-145 (*p* < 0.05) (Figure 6J–6L). Compared with the VSMCs without treatment, Ang II-induced VSMCs treated with NC-mimic and NC-inhibitor plasmids showed increased levels of monocyte colony-stimulating factor (M-CSF), monocyte chemotactic protein-1 (MCP-1), macrophage inflammatory protein-2 (MIP-2), intercellular adhesion molecule 1 (ICAM-1), cleaved caspase-3, NOX4, iNOS, and p47phox along with decreased levels of collagen I and collagen III. These results proved that Ang II could induce high expression of the Egr1-related inflammation genes and injury pathway in cells, thus regulating the oxidative stress and inflammation in cells. Additionally, Ang II-induced VSMCs treated with Egr1 plasmids showed elevated levels of M-CSF, MCP-1, MIP-2, ICAM-1, cleaved caspase-3, NOX4, iNOS, and p47phox whereas decreased levels of collagen I and collagen III. Moreover, Ang II-induced cells treated with si-Egr1 plasmids showed opposite results. Compared with the Ang II-induced cells treated with Egr1 plasmids, Ang II-induced cells treated with Egr1 + miR-145-mimic plasmids displayed reduced levels of M-CSF, MCP-1, MIP-2, ICAM-1, cleaved caspase-3, NOX4, iNOS, and p47phox, along with increased levels of collagen I and collagen III, which were coincident with the results of Ang II-induced cells treated with miR-145-mimic + Sox2ot plasmids (*p* < 0.05) (Figure 6M). These results suggested that the silencing of lncRNA Sox2ot inhibited oxidative stress and inflammation of Ang II-treated VSMCs by binding to miR-145 and subsequently downregulating the Egr1.

### The lncRNA Sox2ot/miR-145/Egr1 axis regulates oxidative stress and inflammation in Ang II-induced AAA mice

With the aforementioned results revealing the roles of the lncRNA Sox2ot/miR-145/Egr1 axis in oxidative stress and inflammation in VSMCs from AAA mice, we further confirmed their role *in-vivo* by injecting the AAA mice with the corresponding recombinant lentiviruses.

**Figure 7:**
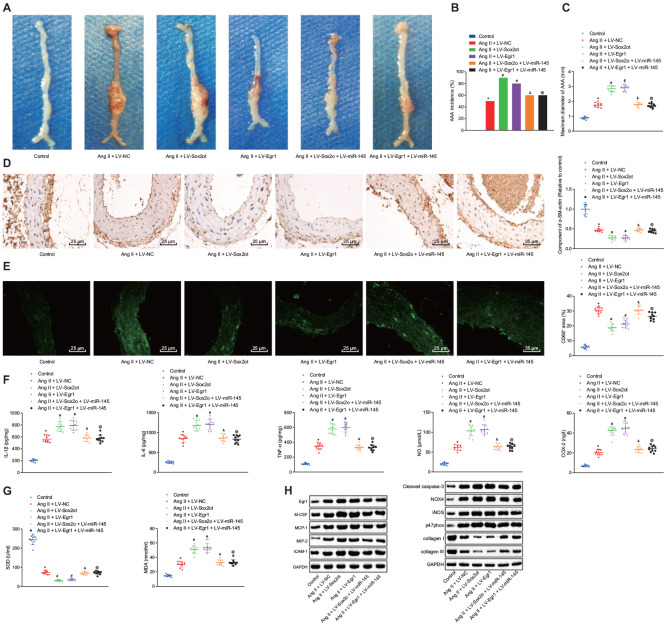
**Silencing of lncRNA Sox2ot affecting miR-145 inhibits oxidative stress and inflammation in Ang II-induced AAA mice by downregulating Egr1.** (**A**) representative morphology images of abdominal aorta specimens from mice; (**B**) incidence of inducing AAA mice; (**C**) the maximum diameter of abdominal aorta from mice; (**D**) α-SM-actin expression in SMCs in abdominal aorta determined using immunohistochemistry (× 400); (**E**) CD68 expression in abdominal aorta detected using immunofluorescence staining (× 400); (**F**) levels of inflammatory factors and oxidative stress-related factors measured in serum of mice measured using ELISA; (**G**) SOD level in serum and MDA levels in abdominal aorta of mice; (**H**) protein levels of Egr1, M-CSF, MCP-1, MIP-2, ICAM-1, cleaved caspase-3, iNOS, p47phox, collagen I and collagen III determined using Western blot analysis; * *p* < 0.05, *vs.* normal mice; # *p* < 0.05, *vs.* AAA mice injected with LV-NC; & *p* < 0.05, *vs.* AAA mice injected with LV-Sox2ot; @ *vs.* AAA mice, injected with LV-Egr1; measurement data were depicted as the mean ± standard deviation; comparisons among multiple groups were analyzed using one-way ANOVA followed by Turkey’s post hoc test; n = 10.

Compared with the AAA mice injected with LV-NC, AAA mice injected with LV-Sox2ot and LV-Egr1 displayed enlarged maximum diameter of abdominal aorta, indicating that lncRNA Sox2ot and Egr1 stimulated the progression of Ang II-induced AAA. However, this effect could be reversed by overexpressing miR-145 (*p* < 0.05) (Figure 7A–7C). Immunohistochemistry and immunofluorescence assay revealed that compared with the AAA mice injected with LV-NC, SMC was decreased and macrophages were accumulated in abdominal aorta from AAA mice injected with LV-Sox2ot and LV-Egr1. Compared with AAA mice injected with LV-Sox2ot and LV-Egr1, AAA mice injected with LV-Sox2ot + LV-miR-145 and LV-Egr1 + LV-miR-145 showed elevated SMC and decreased macrophages (*p* < 0.05) (Figure 7D, 7E). Moreover, ELISA results exhibited that compared with the AAA mice injected with LV-NC, AAA mice overexpressing lncRNA Sox2ot or Egr1 showed increased levels of COX-2, NO, IL-1β, IL-6, TNF-α, and MDA, however, decreased the levels of SOD, which indicated that upregulation of lncRNA Sox2ot and Egr1 stimulated the strength of inflammation and oxidative stress in AAA mice. Compared with LV-Sox2ot- or LV-Egr1-treated AAA mice, these two mice further injected with extra LV-miR-145 individually both displayed reduced levels of COX-2, NO, IL-1β, IL-6, TNF-α, and MDA, yet increased levels of SOD (*p* < 0.05) (Figure 7F, 7G), suggesting that the stimulating effects of lncRNA Sox2ot and Egr1 on inflammation and oxidative stress in AAA mice could be counteracted by overexpression of miR-145. Furthermore, RT-qPCR and Western blot analysis revealed that compared with the AAA mice injected with LV-NC, AAA mice injected with LV-Sox2ot and LV-Egr1 both exhibited increased levels of M-CSF, MCP-1, MIP-2, ICAM-1, cleaved caspase-3, NOX4, iNOS, and p47phox whereas decreased levels of collagen I and collagen III, which verified that upregulation of lncRNA Sox2ot and Egr1 could stimulate inflammation and oxidative stress in AAA mice. Compared with AAA mice injected with LV-Sox2ot and LV-Egr1, AAA mice injected with LV-Sox2ot + LV-miR-145 and LV-Egr1 + LV-miR-145 displayed opposite results (*p* < 0.05) (Figure 7H). Thus, we speculated that silencing of lncRNA Sox2ot affects miR-145 to inhibit the oxidative stress and inflammation in Ang II-induced AAA mice by downregulating Egr1.

## DISCUSSION

AAA is considered a heavy burden on healthcare all over the world [17]. The lncRNAs acting as modulators of miRNAs participate in various physiological processes, particularly, in the progression of cancer [18]. Intriguingly, lncRNAs have been reported to play a role in the regulation of crucial processes in AAA [9], however, the effects of the lncRNAs binding to miRNAs have not been investigated in AAA. In the present study, we aimed to clarify the mechanism of lncRNA Sox2ot in AAA. Our findings revealed that the depletion of lncRNA Sox2ot could affect miR-145 to inhibit the oxidative stress and inflammatory response of VSMCs and results in the suppression of the AAA progression via downregulating the Egr1.

Initial findings from our study showed that the expression of lncRNA Sox2ot and Egr1 was upregulated whereas miR-145 was poorly expressed in VSMCs from AAA mice. Consistently, previous studies have reported the differential expression of about 3688 lncRNAs in AAA patients whilst 1582 lncRNAs of them were upregulated and functionally regulate the development of AAA [9]. Noticeably, LncRNA Sox2ot exhibits high expression in lung cancer [10] and tumor xenografts of breast cancer [11]. Whilst, overexpression of miR-145 has been reported to prevent the occurrence and development of AAA [2], which was according to our findings. Nevertheless, poor expression of miR-145 has been reported in multiple human cancers, such as prostate cancer and gastric cancer [19, 20]. Importantly, Egr1, which is a target gene of miR-145, has been indicated to possess an association with the expression of lncRNA Sox2ot. Moreover, due to its high expression in AAA, Egr1 has been proposed to have potential relevance to human AAA [15, 21]. Collectively, these above-described findings, further support our results, indicating that Egr1 was a target of miR-145 and displayed a positive correlation with lncRNA Sox2ot.

Recently, a handful of lncRNAs have been shown to compete with mRNAs for binding to miRNAs and to contribute to the development of diseases [22]. Consistently, our results confirmed that the upregulation of lncRNA Sox2ot augmented the oxidative stress and inflammation of VSMCs following Ang II induction via suppressing the Egr1 by binding to miR-145 both *in vitro* and *in vivo*, respectively. Moreover, our results exhibited the increased levels of COX-2, NO, IL-1β, IL-6, TNF-α, MDA, cleaved caspase-3, NOX4, iNOS and p47phox whereas reduced levels of SOD, collagen I, and collagen III further support our hypothesis. Additionally, LncRNA RP5-833A20.1 has also been reported to elevate the levels of inflammatory factors, such as IL-1β, IL-6, and, TNF-α in THP-1 macrophages in cardiovascular disease [23]. Besides, inhibition of lncRNA HIF 1 alpha-antisense RNA 1 (HIF1α-AS1) by siRNA can suppress the apoptosis and promote the proliferation of VSMCs by decreasing the caspase-3 expression in thoracic aortic aneurysms [24]. LncRNA Sox2ot knockdown alleviates diabetes mellitus-induced retinal ganglion cell injury by protecting retinal ganglion cells against oxidative stress response [25]. Further studies report that elevated miR-145 can significantly reduce cell growth in gastric cancer [26]. However, a recent study has revealed that miR-145 acts as a critical modulator for VSMC phenotype and proliferation, which represents a promising therapeutic option for vascular diseases [14]. Notably, restoration of miR-145 has been reported to reduce the incidence of AAA, maximum abdominal aortic diameter by inhibiting the MMP2 activation in AngII-infused ApoE-/ mice [2]. Additionally, Egr1 induction has been attributed to the chronic vascular inflammation in AAA as well as other inflammatory vascular diseases due to its promoting effect on the expression of inflammation-related genes [21, 27]. Intriguingly, due to its regulatory role in cell proliferation and apoptosis [27], Egr1, has been reported to reduce the expression of cell proliferation-related genes MMP2 and MMP9 [16]. Egr1 has also been shown to promote the intravascular thrombus formation *in vivo*, contributing to the thrombogenic pathogenesis in human AAA [15].

Accordingly, LncRNA Sox2ot can upregulate the expression of zinc finger E-box-binding homeobox 2 (a target of miR-132) via sponging miR-132 and results in triggering the non-small-cell lung cancer tumor cell proliferation and invasion [28]. Intriguingly, miR-194-5p has been elucidated as a target of lncRNA Sox2ot, however, AKT2 is known to target miR-194-5p. Thereby lncRNA Sox2ot stimulates cell proliferation and metastasis of gastric cancer cells as well as tumor growth and metastasis in nude mice by sponging miR-194-5p and elevating AKT2 [29]. Hence, these abovementioned findings collectively suggest the potential involvement of the lncRNA Sox2ot/miR-145/Egr1 axis in the occurrence and progression of AAA and speculate its potential role in the development of novel therapeutics for AAA.

**Figure 8:**
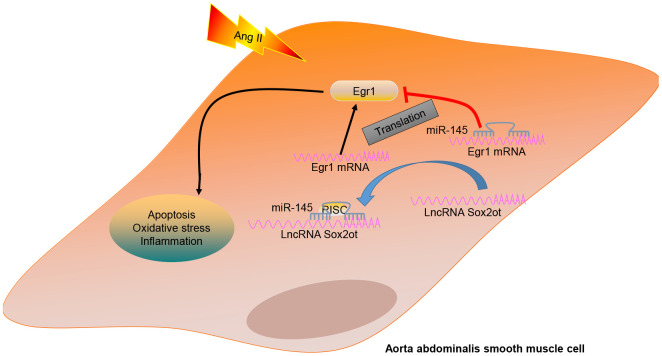
**A mechanism graph of the lncRNA Sox2ot/miR-145/Egr1 axis involved in mice with Ang II-induced AAA.** LncRNA Sox2ot competitively binds to miR-145 and negatively regulates its expression, thus upregulating the expression of Egr1 and then promoting the apoptosis, inflammatory reaction, and oxidative stress of VSMCs, which ultimately accelerates the occurrence of AAA induced by Ang II.

In summary, the current study indicates that depletion of lncRNA Sox2ot could decelerate the occurrence and progression of AAA by serving as a regulator of miR-145 to suppress the proliferation and apoptosis, oxidative stress, and inflammatory reaction of VSMCs in AAA via downregulation of Egr1 (summarized in Figure 8). Thus, the lncRNA Sox2ot-miR-145 network implied a promising perspective for treating patients suffering from AAA.

## MATERIALS AND METHODS

### Ethics statement

All procedures in the animal protocol were reviewed and approved by the Animal Care and Use Committee of Provincial Clinical College of Fujian Medical University, Fujian Provincial Hospital. Extensive efforts were made to ensure minimal suffering of the animals used during the study.

### Establishment of AAA mouse models

A total of 110 ApoE gene knockout C57BL/6 mice (ApoE^-/-^), weighing 17 - 21 g, aged from 5 months, were purchased from Jackson Laboratories (Bar Harbor, ME, USA). Mice fed with a normal chow diet were infused with Ang II (1.44 mg/kg/day) for four weeks using Alzet osmotic pumps (DURECT Corp, Cupertino, CA, USA). Briefly, mice were firstly anaesthetized by a mixture of ketamine (80 mg/kg) and xylazine (5 mg/kg), and subsequently, pumps were implanted subcutaneously in the anaesthetized mice through a small incision in the back of the neck. All incision sites were sutured and healed rapidly without any infection. After finishing infusion with Ang II, their arteries were then subjected to *in vitro* measurement of vascular pathology. Mice were anaesthetized and perfused with phosphate buffered saline (PBS) followed by 4% paraformaldehyde for 10 min via the left ventricle at constant pressure (100 mm Hg). The isolated abdominal aortic arteries were then continued to be fixed with 4% paraformaldehyde for 24 h *in situ*, followed by paraffin embedding. The processed tissues were cut into 7-mm transverse sections for HE staining (Sigma-Aldrich Chemical Company, St Louis, MO, USA). The maximum width of the abdominal aorta was measured using the Image Pro Plus software (Media Cybernetics Inc., Shanghai, China). Aneurysm incidence was defined when an external width of the suprarenal aorta was increased by at least 50% in comparison with that of aortas from the controlled saline-infused mice, which was consistent with the widely adopted clinic criteria to diagnose AAA [30]. Since the average diameter of the normal suprarenal aorta in controlled mice was 0.8 mm, the threshold of 1.22 mm was set as an incidence of aneurysm formation [2].

### Construction of lentivirus expression vectors

miR-145 and its inhibitory expression vectors, lncRNA Sox2ot, Egr1, or negative oligonucleotides, were cloned into an HIV-based vector containing copGFP with the miR precursor under the control of constitutive CMV promoter (System Biosciences, Mountain View, CA, USA). Subsequently, the recombinant lentiviruses: LV-miR-145, LV-miR-145-inhibitor, LV-Sox2ot, LV-Egr1, and LV-NC were constructed. The lentiviruses were produced by transiently transfecting HEK293T cells using SuperFect transfection reagent (Qiagen, Germantown, MD, USA) with three plasmids system (copGFP-miR-145, psPAX2, and pMD2.G). The virus-containing supernatant was collected 72 hours after transfection and filtered through 0.45 mm filters (Millipore, Temecula, CA, USA), and finally preserved at 80°C. Tittering was performed on HEK293 cells using the Adeno-X Rapid Titer kit (BD Biosciences Clontech, Palo Alto, CA, USA) according to the manufacturer’s instructions. Meanwhile, the control lentivirus expressing miR-scr was constructed similarly. Either lentivirus expressing miR-145 or miR-scr was delivered to mice using high-pressure tail vein injection (in 1 mL of PBS over 5 - 10 seconds). A single injection of lenti-miR at a concentration of 10 mg/kg (7.6 × 107 IFU/mouse) was performed on mice one day after the AAA induction [2].

Ten mice from the total 110 mice were randomly selected as the control. The remaining 100 mice were used to establish AAA models, however, among them, 87 mice were successfully modeled The AAA mice were then divided into the following groups: Ang II + LV-miR-NC (miR-145 blank control), Ang II + LV-miR-145 (overexpressed miR-145), Ang II + LV-NC-inhibitor (miR-145-inhibitor blank control), Ang II + LV-miR-145-inhibitor (inhibited miR-145 expression), Ang II + LV-NC (lncRNA Sox2ot/Egr1 blank control), Ang II + LV-Sox2ot (overexpressed lncRNA Sox2ot), Ang II + LV-Egr1 (overexpressed Egr1), Ang II + LV-Sox2ot + LV-miR-145 (overexpressed lncRNA Sox2ot and miR-145), and Ang II + LV-Egr1 + LV-miR-145 (overexpressed Egr1 and miR-145).

### Isolation and culture of VSMCs

Primary VSMCs of mice were obtained and cultured using the method described in a prior study [31]. Briefly, the separated abdominal aorta from 3 successfully modeled mice was washed twice with PBS at 4°C to remove the adipose and connective tissues. Following after, the aorta was cut into 3-mm-thick slices. With enzymatic detachment method, the slices were then incubated with 0.2% collagenase solution at 37°C, after which the VSMCs were isolated from aortic tissue slices, followed by centrifugation at 1000 rpm at 4°C for 15 min. After being washed with PBS, the precipitated VSMCs were inoculated in M231 medium (M231500, Gibco BRL/Invitrogen, CA, USA) with 10% fetal bovine serum (FBS) (10100147, Gibco BRL/Invitrogen, CA, USA), which was supplemented with 100 units penicillin/streptomycin (15140122, Gibco BRL/Invitrogen, CA, USA) per 1 mL medium. The expression of α-SM-actin, a cell marker of VSMCs, was determined by immunofluorescence assay to detect the purity of VSMCs. The VSMCs at passaged three to five were collected for subsequent experiments.

### Establishment of oxidative stress-induced VSMC model and cell treatment

VSMCs (1 × 10^6^ cells) were transfected with 50 μg corresponding overexpression or inhibitor or NC plasmids using 100 μL Lipofectamine^TM^ 2000 transfection reagent (11668019, Invitrogen, Carlsbad, California, USA) according to the instructions. Lipofectamine™ RNA iMAX transfection reagent (13778030, Invitrogen, Carlsbad, California, USA) was used for cell transfection with siRNA. After adding the mixture of transfection reagent and plasmids into the culturing media, cells were incubated at 37°C for 6 hours. After complete medium renewal, the cells were further incubated for 24 - 48 hours and then collected to extract RNA for detecting transfection efficiency. For the oxidative stress-induced VSMC model construction, 2 hours post transfection, VSMCs were incubated with Ang II (10^−6^ M) for 24 hours and their biological characteristics were detected. Then the constructed cell models were further treated with the following conditions: Ang II + NC-mimic (miR-145 blank control), Ang II + miR-145-mimic (overexpressed miR-145), Ang II + NC-inhibitor (miR-145-inhibitor blank control), Ang II + miR-145-inhibitor (inhibited miR-145 expression), Ang II + Sox2ot + NC-mimic (lncRNA Sox2ot/miR-145 blank control), Ang II + Sox2ot + miR-145-mimic (overexpressed lncRNA Sox2ot and miR-145), Ang II + NC (lncRNA Sox2ot/Egr1 blank control), Ang II + Egr1 (overexpressed Egr1 blank control), Ang II + si-NC (silencing Egr1 blank control), Ang II + si-Egr1 (silencing Egr1), Ang II + Egr1 + miR-145-mimic (overexpressed miR-145 and Egr1), Ang II + miR-145-mimic + Sox2ot (overexpressed miR-145 and lncRNA Sox2ot), Ang II + Sox2ot (overexpressed lncRNA Sox2ot), Ang II + si-Sox2ot (silencing lncRNA Sox2ot), and Ang II + miR-145-mimic + NC (overexpressed miR-145 and lncRNA Sox2ot), respectively. The above vectors/plasmids were constructed with the framework of pEGFP-4.1N (Invitrogen, Carlsbad, CA, USA) by Shanghai Sangon Biotechnology Co. Ltd. (Shanghai, China) and siRNA clones were from Sigma Mission RNAi shRNA library.

### RNA isolation and quantitation

Total RNA was extracted using the RNeasy Mini Kit (Qiagen, USA) according to the instructions. All primers used in this study were synthesized by TaKaRa Biotechnology Co., Ltd. (Dalian, China). Following after, total RNA was reverse-transcribed into complementary DNA (cDNA) according to the instructions of miRNA First Strand cDNA Synthesis (Tailing Reaction) kit (B532451-0020, Sangon, Shanghai, China) and cDNA Reverse transcription Kit (RR047A, Takara, Japan). With cDNA as a template, RT-qPCR was performed on an ABI 7500 instrument (Applied Biosystems, Foster City, CA, USA) using SYBR Premix Ex TaqTM II (Perfect Real Time) kit (DRR081, Takara, Japan). The common reverse primer of miRNA and the forward primer of U6 were provided by the miRNA First Strand cDNA Synthesis (Tailing Reaction) kit whereas the remaining primers (Table 1) were synthesized by Sangon Biotechnology Co. Ltd. (Shanghai, China). With U6 and glyceraldehyde-3-phosphate dehydrogenase (GAPDH) serving as the loading control, the expression ratio of the target gene between the experimental and control groups was calculated using the 2^-ΔΔCt^ method.

**Table 1:** Primer sequences for RT-qPCR.

**Target gene**	**Sequences (5'-3')**
miR-145	GUCCAGUUUUCCCAGGAAUCCCU
U6	F: CGCTTCGGCAGCACATATAC
	R: TTCACGAATTTGCGTGTCAT
LncRNA Sox2ot	F: AACTGCTACAAGACAACACC
	R: ATGGTCGCCGCGGGTCCAGCC
Egr1	F: TCCCAGCTCATCAAACCCA
	R: GGCAAACTTCCTCCCACAAA
GAPDH	F: GCCTTCCGTGTTCCTACCC
	R: TGCCTGCTTCACCACCTTC

Note: RT-qPCR, reverse transcription quantitative polymerase chain reaction; GAPDH, glyceraldehyde-3-phosphate dehydrogenase; F, forward; R, reverse.

### Western blot analysis

The total protein was extracted from tissues and cells using radio-immunoprecipitation assay (RIPA) lysis buffer (R0010, Beijing Solarbio Science and Technology Co., Ltd., Beijing, China). After being lysed at 4°C for 15 minutes and centrifuged at 15000 rpm/min for 15 min, supernatants were collected as the lysates and their protein concentration was determined using a bicinchoninic acid (BCA) kit (20201ES76, Yeasen Company, Shanghai, China). Subsequently, the quantified proteins were separated by sodium dodecyl sulfate-polyacrylamide gel electrophoresis (SDS-PAGE) and transferred onto a polyvinylidene fluoride (PVDF) membrane. Afterwards, the membranes were blocked with 5% bovine serum albumin (BSA) at 37ºC for 2 hours and then incubated overnight at 4ºC with the diluted primary rabbit antibodies against Egr1 (ab133695), M-CSF (ab52864), MCP-1 (ab9669), ICAM-1 (ab171123), NOX4 (ab154244), iNOS (ab15323), cleaved caspase-3 (ab13847), p47phox (ab795), collagen I (ab34710), collagen III (ab7778), GAPDH (ab9485), and MIP-2 (M5539, Sigma-Aldrich Chemical Company, St Louis, MO, USA) respectively. The abovementioned antibodies were purchased from Abcam Inc. (Cambridge, MA, USA) except for the MIP-2. After washing with Tris-buffered saline Tween-20 (TBST) for three times (5 min/time), the membrane was incubated with horseradish peroxidase (HRP)-labeled goat anti-rabbit immunoglobulin G (IgG) (ab205718, Abcam Inc.) or donkey anti-goat IgG (ab6566, Abcam Inc.) for 1 hour, followed by 3 TBST washes (5 min/time). At last, the membrane was exposed and developed. Thereafter, the ImageJ 1.48u software (National Institutes of Health, Bethesda, Maryland, USA) was employed for relatively quantifying the corresponding protein of interest by comparing its band intensity and that of the loading control.

### Dihydroethidium (DHE) staining

Intracellular O_2_ concentration was detected using DHE staining. Before staining, the transfected cells were pretreated with either Ang II or PBS. Moreover, DHE (10 μmol/L) was added into the cell culture medium and cells were incubated for another 30 minutes. A Zeiss 4 microscope was employed to observe red fluorescence. A total of 5 to 6 fields of each sample were randomly selected to capture images. The NIH ImageJ Pro software was used to quantify the obtained fluorescence, which was presented as the average fluorescence intensity [5].

### Immunofluorescence staining

Abdominal aortic tissues of mice were fixed in 4% paraformaldehyde, embedded in paraffin, and sliced into 5-μm sections. Then the paraffin in the abdominal aorta slices was removed and these slices were rehydrated. After being fixed with 95% ethanol at -20ºC for 30 min, the treated VSMCs in each group were then blocked with PBS containing 5% BSA at room temperature for 60 min. For detecting the target protein expression, samples were incubated with anti-CD68 primary antibody (1:500; ab53444, Abcam Inc., Cambridge, MA, USA) and anti-α-SM-actin primary antibody (1:50; ab179467, Abcam Inc., Cambridge, MA, USA). Additionally, rabbit IgG was also used here to serve as the NC. Nucleus was stained with 4’6-diamidino-2-phenylindole (DAPI) or 3, 3'-diaminobenzidine (DAB). After incubation with the primary antibodies, the slices were washed with PBS for three times and subsequently incubated with DyLight 549-donkey anti-rabbit (SA5-10064, Invitrogen, Carlsbad, California, USA) supplemented with 5% BSA-PBS under dark conditions at room temperature for 2 hours. Finally, the slices were washed, sealed, and observed under the laser confocal microscope.

### FISH assay

The co-localization of lncRNA Sox2ot and miR-145 was detected using the FISH kit (BIS-P0001, Guangzhou Bersin Biotechnology Co., Ltd., Guangzhou, China). The slide specimens were incubated at 50ºC for 2-3 hours. Then, the slide specimens were taken out and denatured in 2 × sodium citrate buffer (SSC) for 2-3 min followed by dehydration in a series of ethanol, namely 70%, 85%, and 95% for 3 min, respectively. Subsequently, the slide was covered with lncRNA Sox2ot or miR-145 probe hybridization solution labeled by Digoxigenin and the antagonistic lncRNA Sox2ot or miR-145 probe was used as NC. Afterward, slides were cover and the edges were rubber-sealed and processed. After co-denatured in a water bath at 83ºC for 5 minutes, the slides were hybridized at 42ºC for 16 hours. Then, the rubber was removed from the slides and the slides were placed in 2 × SSC until the coverslips detached followed by immersion in 2 × SSC three times with 5 minutes per time. Following after immersion in 70% ethanol for 3 minutes, the slides were naturally dried in the dark, stained with DAPI for 5-10 min, and washed twice with PBS. At last, the slides were photographed under a confocal laser scanning microscope to capture fluorescence images. All images were obtained under the Zeiss LSM880 NLO (2 + 1 with BIG) confocal microscope (Leica Microsystems, Mannheim, Germany).

### HE staining

The abdominal aorta was perfused with normal saline and then fixed with 10% formalin in PBS for 5 minutes under physiological pressure. Then the abdominal aorta was collected, fixed for 24 h, and embedded in paraffin. The cross-section of embedded abdominal aortic tissues was made sure to be 5 mm and then the slices were processed as the conventional HE staining [32].

### CCK-8 assay

Cell viability was evaluated using the CCK-8 kit (CK04, Dojindo, JPN). In brief, VSMCs were seeded into 96-well plates at a density of 1 × 10^4^ cells/well and then cultured for 24 hours for adherence before transfection. Subsequently, 2 hours post-transfection, Ang II or PBS was added into the medium to pretreat the transfected cells for 24 hours. Finally, cells were incubated with CCK-8 reagent (10 μL/well) for 2 hours and the optical density (OD) value was measured at a wavelength of 450 nm using a microplate reader (Bio-Rad Laboratories, Inc., Hercules, CA, USA). Three replicates were set for each group.

### ELISA

Two hours after transfection, Ang II or PBS was added into medium to pretreat the transfected VSMCs for 24 hours. The cell supernatant or separated serum of mice was collected to determine levels of TNF-α (RAB0477; Sigma-Aldrich Chemical Company, St Louis, MO, USA), COX-2 (ab210574, Abcam Inc., Cambridge, MA, USA), IL-6 (RAB0308; Sigma-Aldrich Chemical Company, St Louis, MO, USA), and IL-1β (RAB0274; Sigma-Aldrich Chemical Company, St Louis, MO, USA) by ELISA. Because NO would immediately transform into NO2- and further transform into NO3-, Griess reagent was used to measure the content of NO (KGE001, R&D Systems, Minneapolis, MN, USA) based on the reaction between nitrite and nitrate. Three replicates were set for each group.

### Detection of ROS, MDA, and SOD

VSMCs were seeded into the 96-well plates (5 × 10^3^ cells/well) and incubated at 37ºC for 24 hours for adherence before transfection. Two hours after the transfection, Ang II or PBS was added into medium to pretreat the transfected VSMCs for 24 hours. Then resuspended cells were incubated in pre-heated M231 containing 5 μM of 2,7-Dichlorofluorescein diacetate (H2DCFDA; Gibco, Carlsbad, California, USA) fluorescent probe at 37ºC for 30 minutes, and rinsed twice with PBS. The spectrophotometer (Agilent Technologies, Inc., Palo Alto, CA, USA) was utilized to measure the ROS level at the excitation wavelength of 485 nm and an emission wavelength of 530 nm using the ROS kit (ab113851, Abcam Inc., Cambridge, MA, USA). On the other hand, cell supernatant was collected to determine the MDA level with a thiobarbituric acid method using the MDA kit (ab118970, Abcam Inc., Cambridge, MA, USA) by checking the absorbance at the wavelength of 532 nm. SOD kit (ab65354, Abcam Inc., Cambridge, MA, USA) was used to determine the SOD content in cell supernatant via WST-1, by checking the absorbance at the wavelength of 450 nm.

### RIP assay

The binding of lncRNA Sox2ot and miR-145 to Ago2 protein was detected using a RIP kit (17-701, Merck Millipore, Billerica, MA, USA). Firstly, Protein A coated with antibody was prepared by adding 100 μL of protein A heals to Eppendorf (EP) tubes, after which centrifugation at 4°C was conducted and the supernatant was removed followed by washing with a certain amount of RIP wash buffer. The procedure was repeated three times. The EP tube was added with 5 μg of Ago2 antibody (39854, Active Motif Biotechnology Co., Ltd., Shanghai, China) and rabbit anti-IgG antibody (ab133470, Abcam Inc., Cambridge, MA, USA) followed by incubation with 320 μL RIP wash buffer overnight at 4°C. After adding with 1 mL of washing solution, the samples were centrifuged at 4°C and then the supernatant was discarded. For lysis procedures, HEK 293T cells were collected, washed twice with pre-cooled RIP wash buffer, and centrifuged at 4°C followed by lysing with 200 μL of RIP lysis buffer. The cells were then placed on ice for 2 minutes and centrifuged at 4°C again for 10 minutes. A part of the cell extract (20 μL for each sample) was taken out as inputs, and the other was used for co-precipitation. For co-precipitation, the magnetic bead-antibody complex was added with 900 μL of RIP Buffer and 100 μL of the cell extract for overnight incubation at 4°C to a final volume of 1 mL. Then, the magnetic beads were washed with 0.5 mL of RIP Wash Buffer three times followed by incubation with 150 μL of proteinase K buffer at 55°C for 30 minutes. After twice washes with RIP wash buffer and centrifugation at 4°C, RNA was extracted and purified using the TRIzol method and finally detected by RT-qPCR.

### RNA-pull down assay

The magnetic RNA-Protein Pull-Down kit (20164, Pierce, Rockford, IL, USA) was adopted to detect the binding relation between miR-145 and Egr1. After conventional trypsinization, VSMCs were collected by centrifugation and lysed with RIP lysis buffer on ice for 2 min. After being centrifuged at 4°C for 10 min, the supernatant was divided into several parts (with one part as input) and placed at -80°C. According to kit’s instruction, the miR-145, miR-NC, Egr1-wt, and Egr1-mutant type (mut) were labeled with biotin, enriched with streptavidin magnetic beads, and added with lysis for incubation at 4°C for overnight. At last, the RNA was extracted with TRIzol for purification and finally was detected by RT-qPCR

### Dual-luciferase reporter gene assay

The relationship between miR-145 and Egr1 was predicted using a bioinformatics online prediction website (https://cm.jefferson.edu/rna22/Interactive/) and dual-luciferase reporter gene assay was employed to verify whether Egr1 was indeed a target gene of miR-145. According to the predicted binding sites, the Egr1 mRNA 3'-UTR was inserted into the plasmid pmirGLO (E1330, Promega, Madison, WI, USA) and luciferase vectors, respectively. A mutation site in the seed complementary sequence of Egr1-wt was designed and generated into Egr1-mut reporter plasmid together with Egr1-wt report plasmid. Subsequently, the sequenced luciferase reporter plasmids were co-transfected with miR-145 and miR-NC into the HEK-293T cells. At last, their luciferase activity was detected using the Dual-Luciferase Reporter Gene Assay Kit (E1910, Promega, Madison, WI, USA) on the Glomax20/20 luminometer (E5311, Shannxi Zhongmei Biotechnology Co., Ltd., Xi’an, China).

### Flow cytometry

Apoptosis of VSMCs was detected using Annexin V-fluorescein isothiocyanate (FITC) Apoptosis Detection Kit (Abcam, Cambridge, UK) according to the manufacturer’s instructions. In brief, VSMCs were seeded into a 6-well plate at a density of 1.0 × 10^6^ cells/well, and the processed cells were stained with Annexin V-FITC/propidium iodide (PI) and incubated in the dark at room temperature for 15 minutes. At last, a flow cytometer (FACScan, Beckman Coulter, Inc., Brea, CA, USA) was employed to analyze cell apoptosis.

### Immunohistochemistry

Abdominal aortic tissues of mice were fixed in 4% paraformaldehyde, embedded in paraffin, and sliced into 5-μm-thick sections. Thereafter, the tissue sections were dewaxed in xylene, rehydrated with distilled water followed by antigen retrieval in 0.01 M citric acid buffer at 95°C for 30 minutes. The sections were then incubated with primary antibodies against α-SM-actin and MOMA-2 (ab33451, 1: 50, Abcam Inc., Cambridge, MA, USA), respectively at 4°C overnight. After three washes with PBS containing 0.1% Tween-20 (PBST; #70011-044, Gibco, Carlsbad, California, USA), and treated with 0.3% H_2_O_2_ in TBS for 15 minutes. Subsequently, the sections were incubated with the secondary antibody conjugated with HRP for 4 hours followed by being washed three times and incubated with 140 μL of chromogenic reagent. Finally, the sections were counterstained with hematoxylin. All images were obtained using a computerized microscope (Leica, Berlin, Germany).

### Statistical analysis

All data analyses were conducted using SPSS 21.0 software (IBM Corp. Armonk, NY, USA). All measurement data were presented as mean ± standard deviation. Data with normal distribution and homogeneity of variance between the two groups were compared using unpaired t-test. Comparisons of data among multiple groups were conducted by one-way analysis of variance (ANOVA) followed by Tukey's post hoc tests with corrections for multiple comparisons. Statistical significance was defined when *p* < 0.05.
